# Modeling neuronal consequences of autism-associated gene regulatory variants with human induced pluripotent stem cells

**DOI:** 10.1186/s13229-020-00333-6

**Published:** 2020-05-12

**Authors:** P. Joel Ross, Rebecca S. F. Mok, Brandon S. Smith, Deivid C. Rodrigues, Marat Mufteev, Stephen W. Scherer, James Ellis

**Affiliations:** 1 Department of Biology, University of Prince Edward Island, Charlottetown, PE Canada; 2 Developmental & Stem Cell Biology Program, The Hospital for Sick Children, Toronto, ON Canada; 3 Department of Molecular Genetics, University of Toronto, Toronto, ON Canada; 4 Genetics & Genome Biology Program and The Centre for Applied Genomics, The Hospital for Sick Children, Toronto, ON Canada; 5 McLaughlin Centre, University of Toronto, Toronto, ON Canada

## Abstract

Genetic factors contribute to the development of autism spectrum disorder (ASD), and although non-protein-coding regions of the genome are being increasingly implicated in ASD, the functional consequences of these variants remain largely uncharacterized. Induced pluripotent stem cells (iPSCs) enable the production of personalized neurons that are genetically matched to people with ASD and can therefore be used to directly test the effects of genomic variation on neuronal gene expression, synapse function, and connectivity. The combined use of human pluripotent stem cells with genome editing to introduce or correct specific variants has proved to be a powerful approach for exploring the functional consequences of ASD-associated variants in protein-coding genes and, more recently, long non-coding RNAs (lncRNAs). Here, we review recent studies that implicate lncRNAs, other non-coding mutations, and regulatory variants in ASD susceptibility. We also discuss experimental design considerations for using iPSCs and genome editing to study the role of the non-protein-coding genome in ASD.

## Overview

Autism spectrum disorder (ASD) is a neurodevelopmental disorder with complex genetic underpinnings, and our current understanding of specific genetic risk for ASD comes from the studies of rare mutations affecting DNA that encodes protein-coding exons and genes (for a comprehensive review of the neurobiology and genetics of ASD, see [1]). However, such protein-coding exons represent less than 2% of the human genome, and genome-wide association studies suggest that many ASD-associated variants map to intragenic and intronic regions, as well as non-protein-coding intervals [2]. The recent application of whole-genome sequencing (WGS), which captures the vast majority of chromosomal DNA, has led to the identification of increasing numbers of ASD-associated variants that affect RNA splicing [3–6], long non-coding RNAs (lncRNAs) [7], and transcriptional regulatory elements [4–6, 8–24] (Table 1). However, the functional consequences of non-coding variants are difficult to predict [25] and validate.

**Table 1:** Examples of putative non-coding regulatory variants in ASD

Element/gene	Evidence	Reference
A. Transcriptional regulatory elements		
16q21 near *CDH8* (×2) 3q24 near *C3orf58* (×2)	Rare inherited CNVs affecting non-genic intervals near ASD-associated genes	[13]
5′ UTR of *MBD5*	disruptions of 5′-UTR cause haploinsufficiency of this ASD-associated gene	[14–16]
*EFR3A* promoter	Predicted loss of transcription factor binding (×1)	[4]
Cis regulatory elements of *CTN4*, *LEO1*, *RAF1*, *MEST*	ASD with recurrent variants in intolerant genes paternally inherited deletions of the *LEO1* promoter (×3)	[10]
*NEUROG1* *DLGAP2* *HES1* *FEZF1*	Network differential enrichment analysis: significant neighborhood excess of non-coding variants in Simons Simplex Collection probands and nearby non-coding variants had significant differential effects on activator activities	[6]
regulatory *APBB1*	TADA-A (transmission and de novo association—annotation) analysis: single-nucleotide variants in a regulatory region (×3 (2 conserved))	[2]
near *ARID1B*, *SCN2A*, *NR3C2*, *PRKCA*, *DSCAM*	Disruptive mutations in putative regulatory regions (DNase I hypersensitivity)	[8]
*NR3C2* promoter *DLG2* promoter	Disrupting deletion overlaps functional non-coding regulatory region in the human brain (×1) Same 2.5-kb deletion in the *DLG2* promoter (×3) functional, non-coding regulatory region in developing the human brain	[11]
B. Post-transcriptional regulatory elements		
*miR-873-5p* embedded in *LINGO2* intron	Rare seed mutation	[17]
*CTNND2* and *PTEN* *ALDH5A1*, *GLI2*, *GRIN1*, *KCNH3*, *LAMA2*, and *NISCH*	Splicing/misregulated in genes with neurological phenotypes, increased in ASD	[3]
*SMEK1*	De novo non-coding mutation lying outside of a canonical splice site predicted to disrupt splicing	[6]
*NRXN1*, *TANC2*, *PNPLA7*	Splicing single-nucleotide variants	[2]
C. Long non-coding RNAs		
*AK127244* (2p16.3)	Deletions directly disrupting exonic sequence in ASD (×3) rare inherited CNVs (×5)	[13, 18, 19]
*PTCHD1-AS*	Deletions impacting exons of this gene in multiple males with ASD	[18, 20, 21]
*MSNP1-AS* (5p14.1)	Within ASD GWAS peak, increased expression in the ASD cortex, influences moesin protein levels	[22]
*LINC00689* (7q36.3) *LINC00693* (3p24.1)	Differentially expressed: upregulated in the ASD cortex	[23]
*lnc-NR2F1* (5q15)	Shown to regulate autism risk genes and promote maturation of mouse stem cell-derived neurons	[24]

Although genetically modified rodents can be invaluable model systems to explore functions of ASD-associated protein-coding genes [26], human regulatory elements and non-coding RNAs are not always conserved in mice or rats. Notable interspecies differences have been identified across vertebrates for mechanisms governing the expression of conserved protein-coding genes [27]. Some human regulatory regions are newly evolved or undergo accelerated evolution [28, 29]. Furthermore, among the thousands of known human lncRNAs, nearly one third arose specifically in primate lineages [30]. Together, these observations suggest that human neurons are a more relevant model system for exploring, at least initially, the functions of ASD-associated non-coding variants.

**Fig. 1:**
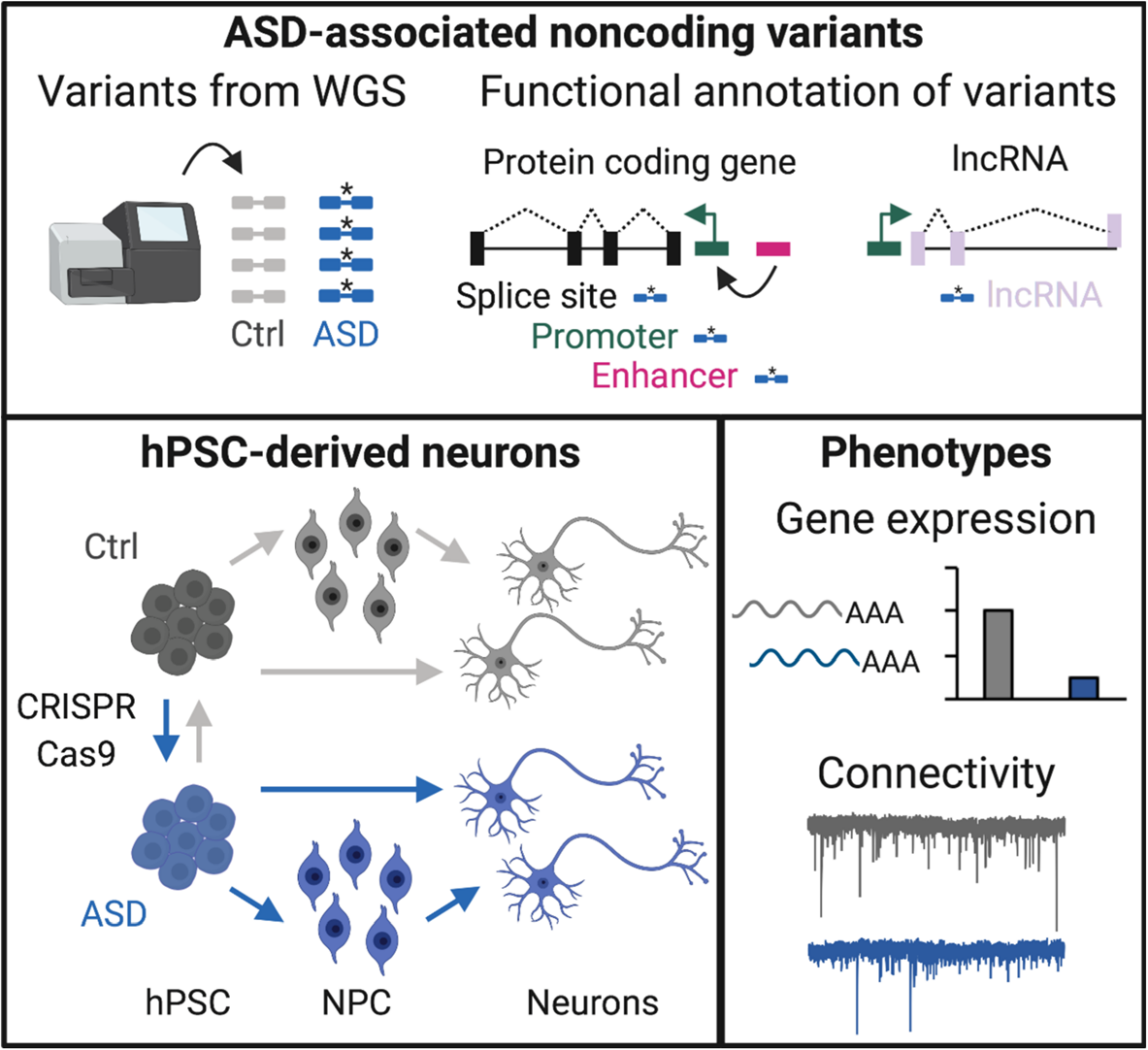
Graphical summary of how hPSC-derived neurons can be used to model the functional consequences of ASD-associated non-coding variants. WGS will identify de novo variants in people with ASD (*), which will then be mapped to specific locations in the genome. Genomic loci are annotated as functional elements based on transcriptomics, chromatin state analyses, and computation predictions. ASD-associated variants can be modeled using personalized iPSCs from people with ASD or by genome editing to introduce or repair ASD-associated variants. hPSC-derived neurons are then made by directed differentiation (via a NPC stage) or direct conversion, and functional consequences of non-coding variants are determined by analyzing gene expression and connectivity

Induced pluripotent stem cells (iPSCs) can produce inexhaustible supplies of personalized neurons that are genetically matched to individuals with ASD or unaffected individuals [31]. CRISPR genome editing has also facilitated the generation of customized neurons with specific variants [32, 33]. iPSC-derived neurons have been used to model ASD, and these studies have consistently implicated altered synaptic function in the underlying pathophysiology of ASD, although the specific mechanisms of synaptic dysfunction vary between models [20, 32, 34–44] (Table 2). Compared to protein-coding genes, experimental perturbation of regulatory elements and non-coding RNAs are more technically difficult, and phenotypic effects may be challenging to detect or interpret [45–47]. We review recent insights into the role of non-coding and regulatory genetic variants in ASD, and we discuss future directions for using human iPSCs and genome editing to explore their functional consequences (Fig. 1).

**Table 2:** Summary of synaptic phenotypes identified in hPSC models of ASD

Gene/locus	Case/ctrl^1^ or isogenic	Cell types	Culture system^2^	Synaptic phenotype^3^		Phenotype rescued?	Ref
				Excit	Inhib^4^		
22q13.3^+/−^	Case/ctrl	Neurons (NPC)	ASD/Ctrl co-culture w/ rt astro	↓ sEPSC freq/ampl; ↓ exc syn#; ↓ AMPAR ampl; ↓ NMDAR ampl	No change	IGF-1 or *SHANK3* transgene	[34]
*TRPC6*^+/−^	Case/ctrl	Neurons (NPC)	Mono-culture	↓ vGLUT1 punctae; ↓ TDL	ND	IGF-1, *TRPC6* transgene, and *TRPC6* agonist	[35]
*NRXN1*^+/−^	Isogenic hESC	Excit neu (Ngn2)	Mono-culture w/ ms astro	↓ mEPSC freq; ↓ release prob; =syn#; =TDL	NA	Not tested	[36]
*STXBP1*^+/−^	Isogenic hESC	Excit neu (Ngn2)	Mono-culture w/ ms astro	↓ mEPSC freq; ↓ release prob; = syn#; =TDL	NA	Not tested	[37]
*SHANK3*^−/−^	Isogenic hESC	Excit neu (Ngn2)	Mono-culture	↓ mEPSC freq; ↓ syn#; ↓ I_h_ channel function	NA	Not tested	[38]
ASD with macrocephaly	Case/ctrl	Neurons (NPC)	Mono-culture	↓ vGLUT1 punctae; ↓ burst freq	ND	IGF-1 (network bursts)	[39]
Not stratified	Case/ctrl	Neurons and astro (NPC)	Reciprocal neu/astro for ASD and Ctrl	↓ exc syn#; ↓ firing rate; (caused by ASD astro)	ND	Culture ASD neurons w/ctrl astro or block IL-6	[40]
10 ASD genes knocked-out with CRISPR	Isogenic iPSCs	Excit neu (Ngn2)	Mono-culture w/ms astro	↓ sEPSC freq (5/10 genes); ↓ burst freq (4/10 genes)	NA	Not tested	[32]
*CNTN5*^+/−^ and *EHMT2*^+/−^	Case/ctrl and isogenic	Excit neu (Ngn2)	Mono-culture w/ms astro	↑ firing rate; ↑ burst freq	NA	Not tested	[42]
*NLGN4*^R704C^	Isogenic hESC	Neurons (Excit: AM; Inhibit: AD)	Mono-culture or co-culture on ctrl lawn w/ ms astro	↑ syn#; ↑ mEPSC freq/ampl	No change	Not tested	[43]
*SHANK2*^+/−^	Case/ctrl and isogenic	Neurons (NPC)	ASD/Ctrl co-culture on ctrl lawn w/ms astro	↑ syn#; ↑ TDL; ↑ sEPSC freq/ampl	ND	Genome editing to repair *SHANK2* and DHPG	[41]
*PTCHD1-AS*^-/y^	Case/ctrl and isogenic	Neurons (NPC)	Mono-culture w/hu or ms astro	↓ AMPAR-mEPSC freq; =exc syn#; =TDL; ↓ NMDAR ampl	ND	Not tested	[20]
9q34^+/−^ or *EHMT1*^+/−^	Case/ctrl and isogenic	Excit neu (Ngn2)	Mono-culture w/rt astro	↓ burst freq; ↑ burst duration =syn#; =TLD; =AMPAR-mEPSC freq/ampl; ↑ NMDAR ampl	NA	MK-801	[44]

^1^*Case/ctrl* iPSC-derived neurons from people with ASD compared to neurons from unaffected controls (ctrl)

^2^*Astro* astrocytes, *rt* rat, *ms* mouse, *hu* human

^3^*Excit* excitatory, *Inhib* inhibitory, *sEPSC* spontaneous excitatory postsynaptic current, *Freq* frequency, *Ampl* amplitude, *exc syn#* excitatory synapse number or density, *AMPAR ampl* amplitude of AMPA receptor-dependent currents, *NMDAR ampl* amplitude of NMDA receptor-dependent currents, *vGLUT1* vesicular glutamate transporter (pre-synaptic marker of excitatory synapses), *TDL* total dendrite length or neurite length, *mEPSC* miniature excitatory postsynaptic current, *release prob* probability of presynaptic neurotransmitter release, *syn#* synapse number or density, *burst freq* network bust frequency (measured by MEA), *firing rate* mean firing rate (measured by MEA), *AMPAR-mEPSC* AMPAR receptor-dependent mEPSC, *burst duration* network bust duration (measured by MEA)

^4^*ND* not determined, *NA* not applicable (*Ngn2* neurons are excitatory)

## Gene regulatory factors in ASD

ASD is now increasingly considered a disorder of synaptic connectivity, and the growing list of ASD-relevant genes has largely converged on two biological processes: synaptic transmission and regulation of gene expression [1, 48, 49]. Known ASD genes involved in gene regulatory processes include transcription factors, RNA-binding proteins, and chromatin regulatory factors, many of which regulate expression of other autism risk genes, and additional regulators of synapse function [1]. ASD-associated chromatin regulators functionally converged upon methylation of H3K4 [50], which is important for the functional demarcation of promoters and enhancers [51]. Recurrent disruption of *writers*, *erasers*, and *readers* of H3K4 methylation in ASD [50] has led to speculation that ASD may be an “enhanceropathy” or a disorder caused by misregulated gene expression [52]. Neuronal gene expression must be finely tuned during development and in response to synaptic activity, so genetic variants that disrupt or alter regulatory elements could have a profound impact on the formation and refinement of synaptic networks [48, 53].

## Non-coding regulatory variants in ASD

Non-coding elements were initially implicated in ASD by analyses of copy number variation (CNV), which identified 15 intergenic loci that were sites of recurrent genomic rearrangement found in ASD subjects [13]. Most of these variants disrupted putative regulatory elements or non-coding RNAs, and several mapped near protein-coding genes that were associated with ASD (*CHD8*, *DIPK2A*/*C3orf58*, and *NRXN1*) or neuronal function (*ASTN2*, *EPH5A*, *SEMA3C*, *UNC5D*). It was initially unknown whether non-coding variants would play a substantial role in ASD, but subsequent WGS studies suggest that approximately 5% of ASD cases may be accounted for by non-coding variants [54]. Also, the determination that non-coding variants largely converge on the same functional processes as ASD-associated protein-coding genes strongly supported the potential for non-coding variants to play causal roles in ASD development [4, 6]. Furthermore, the IQ of people with ASD inversely correlates with the burden of specific RNA regulatory variants, suggesting that non-coding variants may provide novel insights into the clinical heterogeneity of ASD [6]. Relative to exonic variants, it is difficult to predict the functional consequences of non-coding variants [25], although computational tools continue to evolve for prioritization of such variants [2, 4, 6, 12, 55–57]. Several approaches used epigenetic information (e.g., histone marks, chromatin accessibility, and transcription factor binding) from different cell types to computationally predict tissue-specific expression effects of non-coding variants [6, 56, 58]. Interestingly, when tested against known expression quantitative trait loci from the Genotype-Tissue Expression (GTEx) project, one such deep learning approach correctly predicted the directions of expression changes for hundreds of strong effect variants [58]. WGS studies implicated *cis*-regulatory elements like promoters, enhancers, and RNA regulatory sequences in ASD, and many of the genes that are regulated by these elements have been functionally or genetically linked to ASD (Table 1). Next, we highlight recent findings that illustrate the importance of non-coding variants in gene regulation and ASD.

### Transcriptional regulatory elements in ASD

WGS studies have revealed extensive evidence for ASD-associated non-coding variants in transcriptional regulatory elements like promoters and enhancers, which are functionally annotated based on transcriptomics data and chromatin state analyses [59–62]. WGS studies reported that non-coding variants were enriched in conserved enhancers [4] that are accessible in the fetal brain [8] or predicted to regulate ASD genes [5, 8, 12]. WGS has also detected de novo ASD-associated variants in distal promoters (750–2000 bp upstream of transcription start sites), which had a significant association with transcription factor binding sites [9]. Rare recurrent variants were found disrupting predicted promoters for *DLG2* and *NR3C2*, which have both been implicated in brain function or neurodevelopment [11].

Several ASD-associated variants of transcriptional regulatory elements have been shown to directly affect gene expression. For example, one ASD-associated single nucleotide variant mapped to a predicted transcription factor binding site and drove aberrant expression of a reporter gene in the developing mouse forebrain, where the reference sequence was not active [5]. Reporter assays were also used to test transcriptional regulatory properties of variants near known ASD risk genes. Specifically, when 59 prioritized variants from ASD probands were compared to control sequences from their unaffected siblings, 96% of these variants drove significant allele-specific changes in reporter gene expression [6]. Another study reported ASD-associated paternally inherited deletions mapping upstream of the *LEO1* gene, which was previously implicated in ASD by exome sequencing [10]. Interestingly, fibroblast cell lines revealed elevated expression of two neighboring protein-coding genes (*LEO1* and *MAPK6*) in carriers of the deletion who had ASD. The deletions encompass a predicted regulatory element that interacted with the promoters of both *LEO1* and *MAPK6* [10]. This regulatory element is thought to be important for ASD risk because the authors also reported a partially overlapping polymorphic deletion that preserved this element and was common in people who did not have ASD [10]. Together, these studies revealed that ASD-associated variants in non-coding regulatory elements can directly affect the expression of neurodevelopmental genes and ASD risk genes.

### Post-transcriptional regulatory elements in ASD

Misregulation of RNA splicing has also been implicated in ASD (reviewed in [63]), although the underlying genetic mechanisms have not been extensively explored. Alternative splicing is the regulated inclusion or exclusion of specific exons during mRNA processing, which can have a profound impact on protein function. RNA-seq analyses of the brains from individuals with ASD revealed the downregulation of splicing proteins RBFOX1 [64] and nSR100 [65], which were associated with altered splicing of a subset of synaptic genes or genes with conserved microexons (~ 3–15 nucleotides), respectively. RNA-seq analyses also revealed ASD-associated changes in activity-dependent alternative splicing events and regional gene expression patterns in the cortex [23]. Activity-dependent alternative splicing of microexons in *EIF4G1* and *EIF4G3* is impaired in ASD, and deletion of the *Eif4g1* microexon in mice led to prolonged neuronal activation, altered synaptic plasticity, and impaired social interactions [66]. These microexons encode prion-like domains, and their loss led to aberrant translation of proteins that control synaptic transmission and neuronal activity.

WGS studies have begun to uncover ASD-associated non-coding variants in splice signals and untranslated regions. For example, intronic single nucleotide variants that were predicted to alter splicing of transcripts from synaptic genes like *GRIN1* [3], the ASD candidate gene *PTEN* [3], and the neurodevelopmental gene *SMEK1* [6]. WGS studies also reported that ASD-associated de novo variants were enriched in splice sites or untranslated regions (UTRs) of mRNAs [4, 5], particularly UTRs of known ASD risk genes and neurodevelopmental genes [17]. The study of ASD-associated de novo variants in 3’ UTRs of brain-specific transcripts is of particular importance since neuronal mRNAs have the longest 3’ UTRs among all tissues, implying that these molecules are under strong post-transcriptional regulation [67]. Moreover, a few studies reported variants that disrupt untranslated exons of the ASD gene *MBD5* [14–16] (Table 1).

UTRs often harbor binding sites for regulatory RNA-binding proteins and microRNAs (miRNAs) [68], and miRNAs have also been implicated in ASD. miRNAs are short (19–25 nucleotide), non-coding RNA molecules that bind to the UTRs of target mRNAs and affect mRNA stability or translation [69]. miRNAs have been implicated in ASD by genetic studies and animal models, although mechanisms remain largely unexplored. Heterozygous mutation of *AGO1*, which is critical for miRNA function, is associated with a syndromic neurodevelopmental disorder that includes ASD features [70]. Mice with targeted deletions of specific miRNAs or miRNA clusters exhibit pronounced changes in social behavior [71, 72]. The latest release of the miRBase database (v22) accounts for 2654 mature miRNAs identified in humans [73], the majority of which are expressed in the brain [74]. Dozens of microRNAs are consistently misregulated in ASD, and known ASD genes are enriched among targets of these miRNAs [75, 76]. For example, 28 miRNAs differentially expressed in the cerebellar cortexes of people with ASD, 7 of which target the *SHANK3* mRNA [77]. Also, candidate genes for ASD and schizophrenia were enriched for two miRNA target sequences, although the associated miRNAs were not reported [78]. Despite these examples of ASD-associated miRNA misregulation, there are few reports describing causal links between mutations in the UTRs of ASD-risk genes that affect the protein outputs of their respective mRNAs.

Several WGS or whole-exome sequencing studies have reported genetic variants predicted to affect miRNAs or miRNA target sequences. One study specifically tested the hypothesis that ASD-associated synonymous variants in coding sequences may affect miRNA binding sites, although no significant enrichment for miRNA binding sites was detected [79]. The miR-133b/miR-206 cluster was implicated in ASD in a genome-wide association study (GWAS) discovery cohort, although no significant association was detected in a replication cohort [80]. WGS revealed sequence variants that affect the ability of miR-873-5p to bind and regulate ASD-associated target genes, including *NRXN2* and *CNTNAP2* [17]. Together, these findings suggest that ASD-associated variants in post-transcriptional regulatory elements may have the potential to affect splicing, stability, and translation of protein-coding transcripts.

### Long non-coding RNAs in ASD

Most of ~ 16,000 lncRNAs encoded by the human genome [30, 81] have not yet been characterized, but 40% are expressed in the brain and some have been implicated in brain function [82]. For instance, lncRNAs influence neuronal versus glial fate in cortical progenitors [83], while others are upregulated in response to neuronal activity [84–86] and have roles in neuronal excitation and plasticity [87, 88]. When mouse fibroblasts were converted directly to neurons, ~ 60% of differentially expressed transcripts were non-coding RNAs [24], suggesting potential roles in neuronal development.

Data from genetic [18, 20–22, 24] and gene expression studies [23] have also implicated lncRNAs in ASD. The first lncRNA to be convincingly linked to ASD was *PTCHD1-AS* [21], which is frequently disrupted in people with ASD [89]. A recent study supported a role for *PTCHD1-AS* in ASD and also implicated the uncharacterized lncRNA *AK127244* [18, 19]. Genetic and gene expression studies have also suggested potential roles for other lncRNAs in ASD, including *MSNP1-AS* [22], *LINC00689*, and *LINC00693* [23], although the underlying mechanisms for their involvement with ASD remain largely unexplored and could be variable. A recent study reported several other developmentally regulated brain-expressed lncRNAs that are disrupted in ASD or intellectual disability, one of which (*lnc-NR2F1*) was shown to regulate autism risk genes and promote maturation of mouse stem cell-derived neurons [24]. These findings combined with our own recent work on *PTCHD1-AS* (described below) provide direct evidence that ASD-associated non-coding RNAs directly regulate neurodevelopmental processes relevant to ASD.

The aforementioned studies suggest that non-coding variants play important roles in the development of ASD, although the functions of these variants and the regulatory elements they disrupt remain largely unknown. Next, we discuss recent advances in cellular reprogramming and CRISPR technologies that are poised to greatly advance our understanding of ASD-associated non-coding variants.

## Human pluripotent stem cell models of ASD

Human pluripotent stem cells (hPSCs), including human embryonic stem cells (hESCs) and human iPSCs, have the capacity to differentiate into unlimited supplies of brain cells and therefore have tremendous potential for modeling ASD [31]. To date, hPSC studies of ASD have focused largely on variants that disrupt protein-coding genes, which have revealed a wide range of synaptic phenotypes (Table 2). Synaptic phenotyping in iPSC models of ASD has focused primarily on excitatory synaptic function, which is impaired in some models and increased in others (Table 2). The majority of iPSC ASD models with synaptic phenotypes report decreased connectivity, and for some genes, the underlying mechanisms have been determined. Physiological decreases in synaptic function can result from fewer excitatory synapses (*22q13.3*^+/−^, *SHANK3*^−/−^), impairments in neurotransmitter release (*STXBP1*^+/−^, *NRXN1*^+/−^), or hypofunction of excitatory NMDA receptors (*PTCHD1-AS*) (Table 2). Conversely, some other genetic models of ASD show increased synaptic function via increased synapse numbers (*NLGN4*^R704C^, *SHANK2*^+/−^) or hyperfunction of NMDA receptors (*EHMT1*^+/−^) (Table 2). Together, these findings support the notion that increases or decreases in synaptic activity outside of the range that is typical of unaffected individuals may impair sensory processing and social interactions, thereby contributing to ASD development [90].

The protein-coding variants modeled to date are known or predicted to be of high penetrance, whereas the penetrance is unknown for most non-coding variants. Therefore, iPSC experiments modeling of non-coding variants must be carefully designed to minimize heterogeneity and experimental noise. Here, we discuss experimental design considerations for using iPSCs to model ASD, with a specific focus on challenges associated with modeling the consequences of non-coding variants.

### Experimental controls in hPSC models of ASD

Published hPSC models of ASD have used two primary approaches: personalized iPSCs from donors with ASD or genome editing to introduce specific ASD-associated variants in reference lines. Modeling ASD with iPSCs typically uses a case-control model, where iPSC-derived neurons from people with ASD are compared to neurons from people who do not have ASD. These studies are often stratified by gene or shared neurodevelopmental phenotypes (Table 2), and controls are either unaffected family members [20, 41, 42] or unrelated people who are matched to the study subjects by age, sex, or both [20, 34, 35, 39, 40]. This approach is made challenging by extensive intra-individual variability and often requires extensive biological and technical controls/replicates to provide sufficient power to detect neuronal phenotypes [46]. Furthermore, iPSC reprogramming is associated with genomic instability and de novo genomic variants [91, 92], so multiple independent iPSC lines should be assessed for each donor. To overcome genetic and phenotypic heritability among individuals, several groups have increasingly employed “isogenic” genome editing approach for modeling ASD in hPSCs [20, 32, 36–38, 41, 43], which results in mutant and control cell lines with comparable genetic backgrounds.

Isogenic approaches have been extensively used for modeling ASD (Table 2) and are also very powerful when used together with case-control approaches [20, 41, 42]. Genome editing has been used to introduce mutations in several autism candidate genes, which has revealed a range of synaptic and gene expression phenotypes (Table 2). RNA-seq analyses in CRISPR-edited neurons with heterozygous *CHD8* mutations revealed similar misregulated genes in monolayer neurons and organoids [93]. Interestingly, the top misregulated genes in *CHD8*^+/−^ neurons included the lncRNA *DLX6-AS1*, which was also misregulated in organoids from people with idiopathic ASD and macrocephaly [94]. Genome editing approaches have also revealed a wide range of ASD-associated synaptic phenotypes in hESC-derived neurons with engineered variants in *NRXN1*, *STXBP1*, *SHANK3*, and *NLGN4* (Table 2). The penetrance of ASD-associated non-coding variants is largely unknown, so introducing them by genome editing may not result in detectable phenotypes. Starting with iPSCs that are genetically matched to people with ASD improves construct validity and presumably increases the likelihood of detecting phenotypic differences. Upon identification of any ASD-associated phenotypes and correlated gene expression changes, genome editing can be used to repair the non-coding variant or introduce it into control iPSCs. We used this approach to determine that a nonsense mutation in the synaptic gene *SHANK2* was both necessary and sufficient for overconnectivity that we observed in an iPSC model of ASD [41].

Another important consideration for modeling ASD-associated non-coding variants is the selection of controls. Although unaffected family members are often used as controls to partially account for genetic variability [20, 41, 42], some relatives have ASD-like features or carry other combinations of genetic variants that may cumulatively affect synapse function deleteriously [1]. Therefore, we recently reported a panel of iPSC lines from two males and two females who have no overt disease phenotypes and who were also shown by WGS to have minimal loads of genomic variants [95]. These iPSCs were reprogrammed using non-integrating Sendai virus vectors, which is less likely than retrovirus or mRNA reprogramming to result in de novo coding variants [91]. These iPSC lines (Personal Genome Project Canada participants) efficiently differentiate to neurons and other lineages and support genome editing to introduce specific variants. These iPSCs were also analyzed by WGS, which revealed surprising numbers of single nucleotide variants: each iPSC line had over 1000 de novo variants, and genome-edited iPSCs had hundreds of variants compared to the parental line [95]. Some of these de novo variants had the potential to affect disease-related cellular phenotypes, so we identified variant-preferred PGPC iPSC lines for specific applications like neuronal phenotyping. In addition to serving as controls for modeling ASD, we also foresee neurons from the PGPC iPSC lines being very useful for establishing the baseline range of “normal” synaptic functions in human iPSC-derived neurons.

### Neuronal differentiation methods

Although a wide range of protocols has been developed for making specific neuronal and non-neuronal cell types from iPSCs [96], most iPSC approaches for modeling ASD have used neurons with a cortical forebrain identity [31]. Approaches for making iPSC-derived neurons rely primarily on either directed differentiation via a multipotent progenitor stage or direct conversion from pluripotent stem cells to post-mitotic neurons [96]. Both approaches have been used to model ASD (Table 2), although they each have relative advantages and disadvantages.

Phenotypic consequences of regulatory non-coding variants may be restricted to specific lineages or timepoints, and directed differentiation offers the advantage of interrogating multiple cell types within a single experiment. For example, directed differentiation to generate excitatory cortical neurons typically results in mixed cultures that also contain undifferentiated progenitors, inhibitory neurons, and astrocytes [97, 98]. Such mixed cell populations are observed when neurons are differentiated as adherent cultures or in suspension as three-dimensional organoids [99]. Neurons made by directed differentiation also mature asynchronously over the 6–14 weeks required for synapse development, so the resultant cultures contain neurons of varying ages. These mixed populations are advantageous for examining cell fate specification and migration [39] (particularly in organoids [99]) and for exploring interactions between different brain cell types [34, 40]. However, cell type composition in neuronal cultures made by direct differentiation varies between donors and batches, which introduces experimental noise and decreases statistical power to detect phenotypic differences [46, 100].

Direct conversion inherently overcomes heterogeneity by using specific transcription factors to swiftly generate pure populations of post-mitotic neurons [31, 96]. Ectopic expression of human or mouse neurogenin-2 (*NGN2*/*Ngn2*) in PSCs or neural progenitor cells results in homogeneous populations of excitatory cortical neurons that mature in only 3–4 weeks [101, 102]. Neurons made by direct conversion have been used to analyze synapse function and network connectivity in hPSC models of ASD (Table 2), and direct comparison with neurons from directed differentiation have revealed similar phenotypes [36]. Direct conversion can also generate homogenous cultures of inhibitory neurons [103] and astrocytes [104]. Due to their relative homogeneity, neurons made by direct conversion are particularly useful for gene expression analyses to detect regulatory consequences of non-coding variants. However, recent reports suggest that direct conversion may mask ASD-associated phenotypes that arise in the neuronal progenitor phase, such as epigenetic misregulation of synaptic gene expression [105].

As a first step in deciding which approach to use for modeling a potential regulatory variant, it is important to determine when and where the regulatory element is active. Publicly accessible transcriptome data from the developing and adult human brain and from differentiating iPSC-derived neurons [106–112] can be used to determine the cell type and developmental time point at which some regulatory elements are active. Transcription start site and chromatin state data can also be used to infer enhancer activity [60–62, 111–113], which can then be correlated with transcriptome data to identify potential target genes. If variants are predicted to have a function during early brain development or in cell-type specification, then directed differentiation would likely be the more suitable model. Conversely, if variants are predicted to function largely in post-mitotic neurons, then direct conversion may be preferable due to swifter maturation and homogeneous cultures. Upon determining the cell type for experimental analyses, it is of paramount importance that the resultant neurons be tested to ensure that the regulatory element of interest is active and that the cells are suitable for modeling ASD.

## Identifying phenotypes in hPSC models of ASD

### Gene expression analyses

We anticipate that non-coding variants will affect neuronal gene expression, but the design of expression analyses with iPSC-derived neurons can influence the interpretation of results. The heterogeneity that results from directed differentiation is undesirable for transcriptomics [20, 41] and other population-level analyses [98], although specific cell types can be enriched using genetic reporters [114] or cell surface markers [20, 41, 98]. If possible, transcriptomic analyses should be performed using isogenic controls to improve sensitivity to detect expression changes [46]. In our previous work modeling ASD with iPSCs, we found that isogenic pairs revealed robust expression changes [32, 41], whereas an analysis with a case-control design yielded no consistently misregulated genes [20]. It may be particularly difficult to detect expression changes in case-control studies because of the large sample size required to overcome inherent individual and technical variability in iPSC-derived neurons [46, 100]. However, computational advances may improve signal-to-noise for detecting expression changes in mixed populations of neurons, as was recently reported for modeling schizophrenia [115].

Another consideration when modeling transcriptional consequences of non-coding variants in ASD is the need for substantial sequencing depth in transcriptomic analyses. Detection of alternatively spliced exons and low abundance lncRNAs requires thorough read coverage. Indeed, we recently found that a read depth of 60 million paired-end reads per sample was necessary to detect the ASD-associated lncRNA *PTCHD1-AS* [20]. Furthermore, due to the potentially subtle or *cis*-acting effects of non-coding variants on the expression of target genes, it may also be necessary to perform allele-specific gene expression analyses, which also benefits from improved sequencing coverage [116].

### Single-cell analyses: morphology and electrophysiology

iPSC models have confirmed the hypothesis that synaptic dysfunction underlies ASD (Table 2), but the mechanisms underlying synapse dysfunction vary considerably between models. The gold standard approach for assessing excitatory synaptic activity is patch-clamp electrophysiology to record miniature or spontaneous excitatory postsynaptic currents (mEPSCs or sEPSCs, respectively). The frequency of these excitatory synaptic events can be correlated with imaging data that quantify excitatory synapses and dendrite length [37, 38, 41]. Together, these metrics provide insight into the amount of synaptic connectivity and the potential mechanisms underlying any changes. iPSC-derived neurons have revealed a striking dichotomy in ASD-associated synaptic connectivity phenotypes, with different models displaying under- or over-connectivity that can arise by several distinct mechanisms (Table 2).

Some of the phenotypes observed in iPSC-derived neurons recapitulate those observed in mouse models, although contrasting phenotypes have also been reported from these two modeling approaches. Similar functional impairments in excitatory synapses and in hyperpolarization-activated cation (I_h_) channels were observed in human and mouse neurons with *SHANK3* mutations [38]. Human and mouse neurons deficient for *EHMT1* also share decreased network burst frequency and increased NMDA receptor activity [44]. Conversely, heterozygous variants in *STXBP1* [37], *NRXN1* [36], and *SHANK2* [41] are associated with ASD in people and affect connectivity in iPSC models, whereas heterozygous mutations have little or no phenotypic effect on synaptic function in mice. Future work will resolve whether phenotypic differences are species-specific or the result of differences in the cell types that were assessed.

iPSC-derived neurons are subject to both technical and biological variability that can introduce noise in assays of synaptic connectivity. We and others have used co-culture approaches to overcome this variability and record synaptic function in ASD neurons and control neurons within the same cultures [34, 41, 117]. We labeled mature excitatory neurons from controls and from people with ASD using two different fluorescent reporters [41]. These neurons were then sparsely seeded on a lawn of neurons (either from controls or people with ASD) and mouse astrocytes to provide a controlled synaptogenic environment. Simultaneous phenotyping of control neurons and ASD neurons in the same dish revealed increased connectivity in neurons with *SHANK2* variants. This within-well normalization approach reduced experimental variability and improved sensitivity to detect phenotypic changes [41]. An extension of this approach compared isogenic neurons on the lawns of either mutant or control neurons to determine whether any functional impairments were due to pre- or post-synaptic dysfunction [117]. Within-well normalization combined with isogenic controls should reduce noise and improve consistency in human iPSC models of ASD-associated non-coding variants.

Another approach for combatting heterogeneity is to examine gene expression by single-cell transcriptomics. Single-cell RNA-seq has been used to identify misregulated cellular processes in iPSC models of Parkinson’s disease [118] and trisomy 21 [119]. This approach may also be combined with single-cell chromatin accessibility to detect coordinated changes in gene expression and enhancer usage [120]. Finally, single-cell transcriptomics can also be integrated with analyses of neuronal morphology and function using Patch-seq: this modification of patch-clamp electrophysiology uses the patching pipette to deliver a fluorescent dye to reveal neuronal morphology and then to acquire cytoplasmic RNA for single-cell RNA-seq following the completion of recordings [121].

### Network activity

Assessing the function of neural networks can yield insight into how the underlying changes in gene expression, morphology, and synaptic transmission alter connectivity in models of ASD. Using microelectrode arrays (MEAs), neurons can be plated on a grid of microelectrodes to simultaneously record extracellular voltage changes within a synaptic network [122]. Multi-well MEAs can contain several hundred electrodes per plate and read at multiple timepoints to non-invasively acquire information on neural network development and function. Captured signals can be filtered to obtain higher frequency action potential spikes or lower frequency local field potential oscillations [123]. As neurons mature, synchronous network bursting patterns emerge, which can be used to assess differences in network dynamics and circuitry in control and ASD neural cultures. A recent study of 8-month-old cortical organoids even suggested that iPSC-derived neurons can mature to the point of displaying oscillatory network events similar to preterm human electroencephalograph recordings [124]. MEA datasets are also rich in positional information, which can provide insight into circuitry changes resulting from differences in neuron morphology or intrinsic function.

MEAs have recently been used to explore ASD-associated action potential firing and connectivity phenotypes (Table 2), which have revealed further evidence for both under- and over-connectivity in different models. Directed differentiation of mixed excitatory and inhibitory neuron cultures from people with idiopathic ASD exhibited reduced spiking activity and network bursting [39, 40]. Genome-edited excitatory neurons with mutations of several different ASD risk genes had decreased mean firing rate and network burst frequency [32]. Conversely, excitatory neurons from people with heterozygous deletion of *CNTN5* and *EHMT2* exhibited hyperactive networks [42]. iPSC-derived excitatory neurons from individuals with Kleefstra syndrome (who also had ASD diagnoses) showed network bursts with decreased frequency and altered kinetics, and these phenotypes could be rescued pharmacologically [44].

MEA phenotyping is attractive for modeling ASD because the simple non-invasive recordings facilitate higher throughput applications than imaging or patch-clamp electrophysiology. However, a careful experimental design will be necessary to overcome extensive technical and biological variability in baseline MEA metrics (i.e., mean firing rate, number of active electrodes). To overcome this variability, we recommend using isogenic controls when possible, performing ASD/control analyses on the same plate to account for batch effects, and establishing a schedule to ensure consistent latencies between media renewal and MEA recordings. Previous work in hPSC models of ASD has shown that some synaptic phenotypes can be rescued (Table 2). The medium-throughput nature of MEA phenotyping holds promise for building on these rescue approaches and establishing drug-screening platforms to find candidate compounds for correction of under- or over-connected neural networks.

## Functional analyses of non-coding RNA in iPSC-derived neurons

Several recent studies have reported potential roles for miRNAs and lncRNAs in ASD-associated processes like neurodevelopment. Global expression analyses revealed that several miRNAs change in expression during differentiation of iPSC-derived neurons [125, 126]. Expression of miR-4449, miR-181a, and miR-1290 were altered in iPSC models of schizophrenia [127], fragile X syndrome [128], and ASD [129], respectively. miR-199 and miR-214 are upregulated in the neurodevelopmental disorder Rett syndrome, which is associated with impaired neurogenesis in human iPSCs and developing mice [130]. lncRNAs have also been implicated in ASD-associated processes like neurodevelopment and activity-dependent gene expression [53]. The lncRNAs *TUNA* [131] and *lnc-NR2F1* [24] regulate gene expression and neuronal differentiation in mouse embryonic stem cells. *LINC00473* is a primate-specific lncRNA that is robustly induced by synaptic excitation of human iPSC-derived neurons [85] and may regulate the activity-dependent transcription factor CREB [132]. *NEAT1* is a highly abundant lncRNA that is downregulated in response to neuronal depolarization and interacts with epilepsy-associated potassium channels to regulate the excitability of human iPSC-derived neurons [88]. Together, these data suggest that non-coding RNAs contribute to a wide range of ASD-associated neuronal processes.

We recently reported a human iPSC approach for modeling ASD-associated non-coding variants focused on the lncRNA *PTHCD1-AS* [20]. We generated iPSCs from three unrelated males with ASD who had deletions that encompassed one or more exons of *PTCHD1-AS*, along with iPSCs from three unaffected individuals. These iPSCs were differentiated into forebrain neurons, and phenotypic analyses revealed pronounced deficits in excitatory synaptic function, including decreased frequency of mEPSCs and diminished amplitude of NMDA-evoked currents. We also used genome editing to replace a critical exon of *PTCHD1-AS* with a premature polyadenylation sequence, which recapitulated the mEPSC frequency impairment and confirmed the importance of *PTCHD1-AS* in excitatory synaptic function. Our work with *PTCHD1-AS* therefore provides proof of principle that ASD-associated non-coding variants can have pronounced phenotypic consequences in human iPSC-derived neurons.

## Future directions

### CRISPR-based tools for validating regulatory variants

Future analysis of non-coding variants in ASD will benefit from the concurrent application of CRISPR-based tools [133] for artificially manipulating genes and regulatory elements. For instance, variants that alter promoter activity can be independently modeled using CRISPR-interference (CRISPRi) and CRISPR-activation (CRISPRa) to deliver transcriptional repressors or activators to target promoters [133]. In a recent study [117], CRISPRi and CRISPRa were elegantly employed to model the functional consequences of 5 schizophrenia-associated common variants that were previously implicated in misregulation of neuronal genes. CRISPRi and CRISPRa are also potentially useful for functional analyses of lncRNAs, which often have *cis* functions at the endogenous site of lncRNA synthesis [82].

ASD-associated enhancers and splice sites can also be evaluated using CRISPR-based approaches. Enzymatically inactivated Cas9 can be fused to catalytic domains that add or remove histone modifications to directly manipulate enhancer function. For example, Cas9-mediated recruitment of catalytic domains of p300 and HDAC8 has been used to artificially activate or block dynamically regulated enhancers in mouse neurons [134]. CRISPR can also be used to deliver cytidine deaminase to target transcripts to force exon skipping or exon inclusion [135]. These approaches provide the opportunity to recapitulate expression changes caused by ASD-associated non-coding variants, which will independently verify their sufficiency to drive ASD-associated synaptic phenotypes.

### Predicting lncRNA function: detecting cryptic coding capacity

Although lncRNAs are defined in part by their limited protein-coding potential, recent results have challenged the notion that all lncRNAs are devoid of translated open reading frames. Several approaches have been developed for characterizing the translational landscapes of human cells, resulting in the surprising discovery that some lncRNAs are associated with ribosomes and may therefore undergo translation [136]. Historically, open-reading frame prediction algorithms typically have a minimum threshold of 100 codons, leading some transcripts that encode small proteins or peptides to be classified as lncRNAs [137]. However, ribosome profile sequencing (Ribo-seq) has revealed that some of these lncRNAs have sequencing reads with 3 nucleotide periodicity, as is seen in normal ribosome movement on coding mRNAs [136]. Furthermore, Ribo-seq-enriched lncRNAs often encode conserved short open reading frames that are enriched in synonymous mutations [136, 137]. In a recent translational profile of human heart, 22% of expressed lncRNAs were translated into potential micropeptides [138]. Therefore, future studies of ASD-associated lncRNAs should first seek to rule out peptide/protein-coding potential before attempting to model any non-coding regulatory functions.

## Conclusions

Continued WGS will invariably lead to increasing numbers of ASD-associated non-coding variants being discovered. iPSCs and genome editing provide exciting opportunities to model the consequences of these variants in human neurons and for correlating gene expression changes with functional differences in synaptic connectivity. Careful experimental design and use of well-selected experimental controls (including isogenic controls when possible) will reduce experimental noise and heterogeneity, leading to more sensitive analyses. Determination of the phenotypic consequences of non-coding variants will provide insights into both the neuronal dysfunction that underlies ASD and the mechanisms governing the regulation of human genetic information.

## Data Availability

Not applicable
